# Correcting the Actual Reproduction Number: A Simple Method to Estimate *R*_0_ from Early Epidemic Growth Data

**DOI:** 10.3390/ijerph7010291

**Published:** 2010-01-21

**Authors:** Hiroshi Nishiura

**Affiliations:** 1 PRESTO, Japan Science and Technology Agency, Saitama, 332-0012, Japan; 2 Theoretical Epidemiology, University of Utrecht, Yalelaan 7, Utrecht, 3584CL, The Netherlands; E-Mail: h.nishiura@uu.nl; Tel.: +31-30-253-4097; Fax: +31-30-252-1887

**Keywords:** transmission, infectious diseases, basic reproduction number, epidemiology, statistical model, estimation techniques, HIV, AIDS

## Abstract

The basic reproduction number, *R*_0_, a summary measure of the transmission potential of an infectious disease, is estimated from early epidemic growth rate, but a likelihood-based method for the estimation has yet to be developed. The present study corrects the concept of the actual reproduction number, offering a simple framework for estimating *R*_0_ without assuming exponential growth of cases. The proposed method is applied to the HIV epidemic in European countries, yielding *R*_0_ values ranging from 3.60 to 3.74, consistent with those based on the Euler-Lotka equation. The method also permits calculating the expected value of *R*_0_ using a spreadsheet.

## 1. Introduction

### 1.1. The Basic Reproduction Number

The basic reproduction number, *R*_0_, of an infectious disease is the average number of secondary cases generated by a single primary case in a fully susceptible population [1]. *R*_0_ is the most widely used epidemiological measurement of the transmission potential in a given population. Statistical estimation of *R*_0_ has been performed for various infectious diseases [2,3], aiming towards understanding the dynamics of transmission and evolution, and designing effective public health intervention strategies. In particular, *R*_0_ has been used for determining the minimum coverage of immunization, because the threshold condition to prevent a major epidemic in a randomly-mixing population is given by 1-1/*R*_0_ [4]. In addition, *R*_0_ gives an estimate of the so-called final size, *i.e.*, the proportion of the population that will experience infection by the end of an epidemic [5,6].

### 1.2. Statistical Estimation of R_0_

Methodological discussions concerning the statistical inference of *R*_0_ are still in progress, and it is recognized that the estimate is very sensitive to dispersal (or underlying epidemiological assumptions) of the progression of a disease [7]. When one estimates *R*_0_ using epidemic data of an emerging (or exotic) disease, the exponential growth rate, *r*, of infections during the initial phase of the epidemic is used [8,9]. Assuming that the generation time, *i.e.*, the time from infection of a primary case to infection of a secondary case generated by the primary case [10], is known (or separately estimated from other empirical data), the growth rate *r* is transformed to *R*_0_ using that knowledge (see below). That is, the conventional estimation technique has required two statistical steps, namely, first estimate *r* and then convert *r* to *R*_0_.

The estimation method can be illustrated by employing a simple renewal process which adheres to the original definition of *R*_0_ [1]. Let *j*(*t*) be the number of new infections (*i.e.*, incidence) at calendar time *t*. Supposing that each infected individual on average generates secondary cases at a rate *A*(*τ*) at time *τ* since infection (where *τ* is referred to as the “infection-age” hereafter), *j*(*t*) is written as:

(1) $$j(t)=\int_{0}^{\infty }A(\tau )j(t-\tau )d\tau .$$

Since *R*_0_ represents the total number of secondary cases that a primary case generates during the entire course of infection, the estimate is given by ([11]):

(2) $$R_{0}=\int_{0}^{\infty }A(\tau )d\tau .$$

When *j*(*t*) follows an exponential growth path, it is easy to extract the integral of *A*(*τ*) from equation (1). Supposing that the incidence grows exponentially at a rate *r*, we have *j*(*t*) = *k*exp(*rt*) where *k* is a constant, and moreover, *j*(*t* – *τ*) = *k*exp(*rt*)exp(–*rτ*). This simplifies (1) to the so-called Euler-Lotka equation:

(3) $$1=\int_{0}^{\infty }A(\tau )\;\text{exp}(-r\tau )d\tau .$$

Since the density function of the generation time, *g*(*τ*), represents the frequency of secondary transmissions relative to infection-age *τ*, we can write *g*(*τ*) as:

(4) $$g(\tau )=\frac{A(\tau )}{\int_{0}^{\infty }A(s)ds}=\frac{A(\tau )}{R_{0}}.$$

Replacing *A*(*τ*) in the right-hand side of (3) by that of (4), an estimator of *R*_0_ is obtained [9]:

(5) $${\hat{R}}_{0}=\frac{1}{\int_{0}^{\infty }g(\tau )\;\text{exp}(-r\tau )d\tau }.$$

The estimation of *R*_0_ is achieved by measuring the exponential growth rate *r* from the incidence data and also by assuming that *g*(*τ*) is known (or separately estimated from empirical observation such as contact tracing data [12,13]). It should be noted that the above mentioned framework has not been given a likelihood-based method for estimating *R*_0_ (*i.e.*, a likelihood function used for fitting a statistical model to data, and providing estimates for *R*_0_, has been missing). Moreover, equation (5) may not be easily used by non-experts, e.g., an epidemiologist who wishes to estimate *R*_0_ using her/his own data.

The purpose of the present study is to offer an improved framework for estimating *R*_0_ from early epidemic growth data, which may be slightly more tractable among non-experts as compared with the above mentioned estimator (5). A likelihood-based approach is proposed to permit derivation of the uncertainty bounds of *R*_0_. For an exposition of the proposed method, the incidence data of the HIV epidemic in Europe is explored.

## 2. Methods

### 2.1. Actual Reproduction Number

In addition to *R*_0_, a different measurement of the transmission potential using widely available epidemiological data, the actual reproduction number, *R*_a_, has been proposed for HIV/AIDS [14]. The concept of *R*_a_ is much simpler than *R*_0_ in that *R*_a_ is defined as a product of the mean duration of infectiousness and the ratio of incidence to prevalence [15]. The prevalence *p*(*t*) at calendar time *t* is written as:

(6) $$p(t)=\int_{0}^{\infty }\Gamma (\tau )j(t-\tau )d\tau$$

where *Γ*(*τ*) is the survivorship of infectiousness, or probability of being infectious, at infection-age *τ*. Letting *β*(*τ*) be the transmission rate, which depends primarily on the frequency of contact and infectiousness at infection-age *τ*, the above mentioned *A*(*τ*), the rate of secondary transmission per single primary case at *τ*, under an assumption of a Kermack and McKendrick type model, is decomposed as ([16]):

(7) A(τ)=β(τ)Γ(τ).

The actual reproduction number *R*_a_ is written as:

(8) $$R_{a}=\frac{j(t)}{p(t)}D=\frac{\int_{0}^{\infty }\beta (\tau )\Gamma (\tau )j(t-\tau )d\tau }{\int_{0}^{\infty }\Gamma (\tau )j(t-\tau )d\tau }D.$$

where *D* is the mean generation time (or what was previously described as the mean duration of infectiousness [14,15]). If the transmission rate *β*(*τ*) is constant *β* (*i.e.*, independent of infection-age), *R*_a_ = *βD*. Moreover, from equation (2)*R*_0_ is given by:

(9) $$R_{0}=\int_{0}^{\infty }\beta (\tau )\Gamma (\tau )d\tau =\beta D.$$

*R*_a_ coincides with *R*_0_ as long as *β*(*τ*) is constant. Nevertheless, in many instances, the contact frequency and infectiousness (which may be partly reflected, for example, in the viral load distribution of the infected host) vary as a function of infection-age *τ*. The variation in *β*(*τ*) is particularly the case for HIV infection. Thus, although the usefulness of the incidence-to-prevalence ratio and *R*_a_ has been emphasized to have an application in HIV/AIDS [15], *R*_a_ tends to yield a biased estimate (if *R*_a_ is regarded as a proxy for *R*_0_), and the estimate of *R*_a_ is not as robust as that is obtained with equation (5) to objectively interpret the transmission potential [17,18].

### 2.2. Correcting R_a_

**Figure 1.**
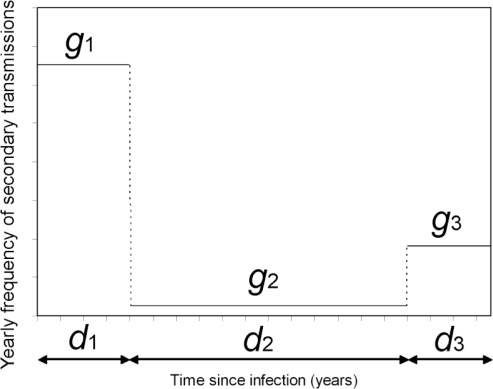
The relative frequency of secondary transmissions of HIV as a function of the time since infection. A step function is employed to approximately model the frequency of secondary transmissions relative to infection-age. For *d*_1_ years shortly after infection, the frequency *g*_1_ is very high. Subsequently, for *d*_2_ years (*i.e.*, during the asymptomatic period), *g*_2_ is persistently low, followed by a time period with high infectiousness *g*_3_ for *d*_3_ years until death or no secondary transmission due to AIDS. Following a statistical study [20], *d*_1_, *d*_2_ and *d*_3_ are assumed to be 0.24, 8.38 and 0.75 years. Assuming that the contact frequency does not vary as a function of time since infection, *g*_1_, *g*_2_ and *g*_3_ are estimated at 1.30, 0.05 and 0.36 per year.

Here, the above mentioned negative aspect of *R*_a_ is reconsidered by correcting the definition of *R*_a_. The disease of interest in the present study is HIV. The frequency of secondary transmissions relative to infection-age *τ* (*i.e.*, the generation time distribution), approximated by a step function, is shown in Figure 1. As has been known for some time [19], the frequency of secondary transmissions is very high shortly after infection (for a duration *d*_1_ = 0.24 years), followed by a long asymptomatic period with a low frequency of secondary transmissions (for *d*_2_ = 8.38 years) [20]. Subsequently, the frequency rises sharply again resulting in a substantial number of secondary cases for a duration *d*_3_ = 0.75 years until death or until the infected individual ceases risky sexual contact due to AIDS [20–22]. Assuming that the contact frequency does not vary as a function of infection-age, *g*_1_, *g*_2_ and *g*_3_ have been estimated at 1.30, 0.05 and 0.36 per year [20]. Here, *g*(*τ*) is the density function of the generation time, with a mean of 3.79 years.

Here the concept of *R*_a_ is corrected. The equation (8) is rewritten as:

(10) $$R_{a}(t)=\frac{j(t)}{\frac{1}{D}\int_{0}^{\infty }\Gamma (\tau )j(t-\tau )d\tau }$$

The numerator represents the number of new infections at calendar time *t*, while the denominator was originally intended to represent “the total number of infectious individuals” who can potentially be primary cases with an equal chance at time *t*. Nevertheless, in order that the estimator of the actual reproduction number coincides with that of *R*_0_, the concept of the denominator is better replaced by “the total number of effective contacts (which can potentially lead to secondary transmissions with an equal probability)”. Therefore, the corrected *R*_a_ is better written as:

(11) $$R_{a}^{'}=\frac{j(t)}{\int_{0}^{\infty }g(\tau )j(t-\tau )d\tau }.$$

where *g*(*τ*), in the renewal equation with the Kermack and McKendrick type assumption, is written as the normalized density of secondary transmissions, *i.e*.,:

(12) $$g(\tau )=\frac{\beta (\tau )\Gamma (\tau )}{\int_{0}^{\infty }\beta (s)\Gamma (s)ds}=\frac{\beta (\tau )\Gamma (\tau )}{R_{0}}$$

Replacing *g*(*τ*) in the right-hand side of (11) by that of (12), we get:

(13) $$R_{a}^{'}=\frac{j(t)}{\frac{1}{R_{0}}\int_{0}^{\infty }\beta (\tau )\Gamma (\tau )j(t-\tau )d\tau }=R_{0}.$$

Thus, the estimator of corrected *R*_a_ in (11) is identical to that of *R*_0_. In other words, *R*_0_ can be estimated from the incidence data and the generation time without assuming exponential growth of cases during the early phase of an epidemic. It should be noted that the ratio of prevalence to mean generation time *p*(*t*)/*D* in the denominator of the right-hand side of (10) has been replaced by “the total number of effective contacts that have equal potential to generate secondary cases”.

### 2.3. Data

**Figure 2.**
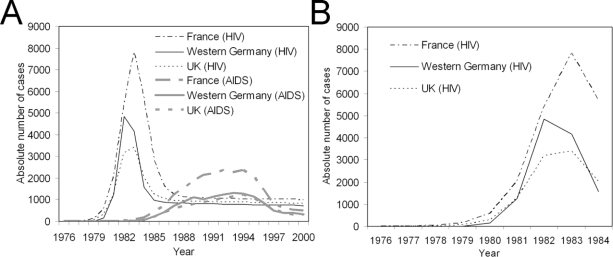
Epidemic curves of HIV/AIDS in France, Western Germany and the United Kingdom (UK) from 1976–2000. **A.** The yearly number of new HIV infections (*i.e.*, incidence) and new AIDS cases from 1976–2000. AIDS cases are the observed data, while HIV incidence is estimated by means of a back-calculation method used by Artzrouni [23]. **B.** The early growth phase of the HIV epidemic. The peak incidence was observed in 1982 in Western Germany and 1983 in France and the UK.

Here the epidemic data of HIV/AIDS in three European countries: France, the Western part of Germany (*i.e.*, the former Western Germany) and the United Kingdom (UK) are investigated [23]. Figure 2A shows the yearly incidence in these countries from 1976–2000. During the observation period, a total of 23,243, 13,126 and 11,491 AIDS cases were diagnosed in France, Western Germany and the UK, respectively. Although the time of HIV infection is not directly observable, the HIV incidence has been estimated by employing a back-calculation technique and using the AIDS incidence and the incubation period distribution of AIDS [23]. The present study does not discuss the details of back-calculation, but explanatory guides can be found elsewhere [24–26]. Figure 2B shows the enlarged HIV incidence curve during the initial phase of an epidemic. The peak incidence was observed in 1982 for Western Germany and 1983 for France and the UK. In the following, the time period from 1976 up to one year prior to the peak incidence is taken as the early growth phase. For the purpose of an exposition of the proposed method, the HIV incidence is assumed to have been in a single homogeneously-mixing population.

### 2.4. Statistical Analysis

*R*_0_ is estimated using two different methods, one employing the estimator (5) and another using the corrected *R*_a_. For the former approach, the exponential growth rate is estimated via a pure birth process [27]. Given that the cumulative incidence from year 0 to *t* – 1 is observed, the conditional likelihood of observing the cumulative incidence *J*_t_ cases in year *t* is proportional to ([28]):

(14) $$\text{exp}\left(-r\sum_{i=0}^{t-1}J_{i}\right){\left(1-\text{exp}(-r)\right)}^{J_{t}-J_{0}},$$

from which the maximum likelihood estimate and the 95% confidence intervals (CI) of *r* (per year) are obtained. Given a fixed generation time distribution *g*(*τ*), the uncertainty bound of *R*_0_ mirrors the uncertainty in the estimate of *r*. The translation of *r* into *R*_0_ via (5) is made by using *g*(*τ*) in Figure 1.

The latter method, proposed in the present study, employs the estimator of corrected *R*_a_ in (11). Since the data are yearly, the equation (11) needs to be rewritten in discrete-time:

(15) $${\hat{R}}_{0}=\frac{j_{t}}{\sum_{s=0}^{\infty }g_{s}j_{t-s}}$$

where the discrete density function of the generation time, *g*_s_ is assumed to be given by *g*_s_ = *G*(*s* + 1) – *G*(*s*), where *G*(*s*) is the cumulative distribution function of the generation time of length *s*, but *g*_0_ is calculated as a normalized yearly average, because *d*_1_ is as short as 0.24 years.

The likelihood of estimating *R*_0_ with (15) is proposed as follows. First, the inverse of both sides of (15) is taken:

(16) $$\frac{1}{R_{0}}=\frac{\sum_{s=0}^{\infty }g_{s}j_{t-s}}{j_{t}}$$

The numerator of the right-hand side indicates the total number of effective contacts made by potential primary cases in year *t* that have an equal probability of resulting in a secondary transmission, and the denominator is the number of secondary cases in year *t*.

**Figure 3.**
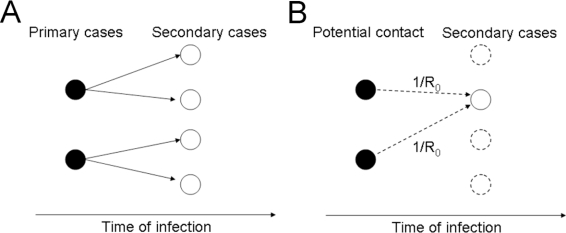
The transmission tree with *R*_0_ = 2. (A) Black circles represent primary cases that are infectious to others at time *t* and white circles are secondary cases generated by the primary cases. Secondary transmissions from primary to secondary cases are given with the basic reproduction number, *R*_0_ = 2, *i.e.*, each primary case generates two secondary cases. (B) Reconstruction of the transmission tree. Given that all the potential contacts made by primary cases (black circles) are known using the incidence data and the generation time distribution, the probability that each potential contact resulted in a secondary transmission is given by 1/*R*_0_.

The right-hand side of equation (16) is interpreted as follows. Figure 3A shows a transmission tree, *i.e.*, a representation of who infected whom, where each primary case on average generates two secondary cases. A transmission tree of this kind is usually unobserved unless rigorous contact-tracing with microbiological examination is implemented. Thus, a likelihood-based approach to reconstructing the tree is considered (Figure 3B) [29–31]. Given the total number of effective contacts that have equal potential for resulting in secondary transmission, the probability of a single effective contact resulting in a secondary transmission (or the probability that a secondary case is linked to an effective contact made by a single primary case in year *t*) is given by 1/*R*_0_. This is a simple binomial sampling process. In other words, the likelihood function for estimating *R*_0_ is:

(17) $$L(R_{0})=\prod_{t=1}^{T}\left(\begin{array}{c} j_{t}\\ \sum_{s=0}^{\infty }g_{s}j_{t-s} \end{array}\right){\left(\frac{1}{R_{0}}\right)}^{\sum_{s=0}^{\infty }g_{s}j_{t-s}}{\left(1-\frac{1}{R_{0}}\right)}^{j_{t}-\sum_{s=0}^{\infty }g_{s}j_{t-s}}$$

where *T* is the most recent time point of observation within an early (linear) epidemic growth stage. The maximum likelihood estimate of *R*_0_ is obtained by minimizing the negative logarithm of (17), and the 95% CI are derived from the profile likelihood.

## 3. Results and Discussion

Table 1 shows the estimates of *r* and *R*_0_ for HIV in France, Western Germany and the UK. The maximum likelihood estimates of *r* ranged from 1.15 to 2.15 per year with the smallest estimate in France and the highest in Western Germany. The 95% CI for Western Germany did not overlap with those of the other two countries, reflecting the steep rise in incidence in this region in Figure 2B. Based on the exponential growth assumption in (5), the estimate of *R*_0_ ranged from 3.65–4.08. Again, Western Germany yielded the highest estimate without an overlap of the uncertainty bound with the other two countries. The maximum likelihood estimate of *R*_0_ based on the proposed new method ranged from 3.59 to 3.74. Not only were the qualitative patterns for the expected values of *R*_0_ consistent with those based on an exponential growth assumption, but the 95% CI also broadly overlapped with those based on the other method. In particular, although *R*_0_ in Western Germany using the proposed method is smaller than that based on an exponential growth assumption, the 95% CIs of the two methods overlapped. The 95% CI based on the proposed method appeared to be wider than those based on exponential growth assumption. Since HIV is mainly transmitted via sexual contact, the above mentioned estimate may vary with the mixing pattern and contact frequency (thus, there is no general disease-specific *R*_0_, especially for HIV/AIDS). At least, compared with a previous estimate of *R*_0_ as ranging from 13.9 to 54.5 in the USA, based on an exponential growth assumption that adopted a mean infectious period of 10 years [32], *R*_0_ in the present study appeared to be much smaller using a precise estimate of the generation time distribution.

The present study proposed the use of the corrected actual reproduction number, *R*_a_, for statistical inference of *R*_0_ based on incidence and known relative frequency of secondary transmissions (*i.e.*, the generation time distribution) during the early growth phase of an epidemic. The previously available method using (5) forced us to adopt an exponential growth assumption, and moreover, an additional step towards the estimation of *r* (*i.e.*, the translation of *r* to *R*_0_) was required [9]. The proposed method does not necessarily require an exponential growth assumption and provides a “short-cut” to estimate *R*_0_ from incidence data. The simple likelihood function employing a binomial distribution was also proposed to yield an appropriate uncertainty bound of *R*_0_. It should be noted that given the knowledge of *g*_s_ and readily available incidence data, equation (15) permits calculation of the expected value of *R*_0_ without likelihood. Such a calculation can be attained using any spreadsheet.

The usefulness of the actual reproduction number, calculated as a product of the mean generation time and the incidence-to-prevalence ratio, has been previously emphasized in assessing the epidemiological time course of an epidemic [14,15]. However, it appears that *R*_a_ does not precisely capture the secondary transmission if the transmission rate *β*(*τ*) varies with infection-age *τ* [18], and moreover, the cohort- and period-reproduction numbers directly derived from the renewal equation have been suggested to be more accurate figures in capturing the underlying transmission dynamics [17,33]. In the present study, replacing the denominator (*i.e.*, prevalence) of *R*_a_ by the total number of potential contacts, it was shown that the *R*_0_ derived from the renewal equation coincides with the corrected actual reproduction number, *R*_a_, and also that the likelihood can be quite easily derived. The corrected *R*_a_ does not require prevalence data, and uses only the incidence data and the generation time distribution.

Many future tasks remain, however. Most importantly, the estimation of *R*_0_ from early epidemic growth data for a heterogeneously-mixing population is called for. *R*_0_ in the present study can even be interpreted as *R*_0_ for a heterogeneously-mixing population (*i.e.*, the dominant eigenvalue of the next-generation matrix), provided that the early growth rate is the same among sub-populations (though it is not the case if the growth rate varies across sub-populations) [34,35]. Analyzing heterogeneous transmission among approximately-aggregated discrete groups, the estimate of the next-generation matrix would give more detailed insights into the epidemic dynamics, including the most important target host for intervention [36]. As discussed in a modeling study in this special issue of the *International Journal of Environmental Research and Public Health* [37], understanding the implications of sexual partnerships and their variations as a function of calendar time as well as infection-age is also of utmost importance. As the next step for a similar estimation framework, methods for estimating robust *R*_0_ and the next-generation matrix from structured data (*i.e.*, data stratified by age- and/or risk-groups) will be useful. Despite the future challenges, I believe the present study at least satisfies a need to offer a likelihood-based approach to estimate *R*_0_ from early epidemic growth data, while being easily tractable and calculable among general epidemiologists.

## Figures and Tables

**Table 1.** Comparison of the estimates of the basic reproduction number for HIV/AIDS obtained using two different estimation methods.

**Country**	***r* (/year)^1^**	***R*_0_ (exponential growth) ^2^**	***R*_0_ (proposed likelihood) ^3^**
France	1.15 (1.12, 1.17)	3.65 (3.64, 3.66)	3.59 (3.38, 3.81)
Western Germany	2.15 (2.02, 2.29)	4.08 (4.02, 4.14)	3.74 (3.43, 4.08)
UK	1.21 (1.18, 1.25)	3.67 (3.66, 3.69)	3.65 (3.38, 3.96)

1 The intrinsic growth rate during the exponential growth phase;

2 the basic reproduction number estimated using equation (5);

3 the basic reproduction number estimated using equation (17); the 95% confidence intervals are shown in parentheses.
